# Efficient treatment of Parkinson’s disease using ultrasonography-guided rhFGF20 proteoliposomes

**DOI:** 10.1080/10717544.2018.1482972

**Published:** 2018-07-25

**Authors:** Jianlou Niu, Junjun Xie, Kaiwen Guo, Xiaomin Zhang, Feng Xia, Xinyu Zhao, Lintao Song, Deli Zhuge, Xiaokun Li, Yingzheng Zhao, Zhifeng Huang

**Affiliations:** School of Pharmaceutical Sciences & Center for Structural Biology, Wenzhou Medical University, Wenzhou, Zhejiang, China

**Keywords:** FGF20, Parkinson’s disease, focused ultrasound, liposome

## Abstract

Fibroblast growth factor-20 (FGF20) is a paracrine member of the FGF family that is preferentially expressed in the substantia nigra pars compacta (SNpc). Previous studies have demonstrated that FGF20 enhances the survival of dopaminergic neurons suggesting the potential use of FGF20 to treat Parkinson’s disease (PD). However, the reduced solubility of the bacterial recombinant human FGF20 (rhFGF20) and the absence of efficient strategies to transport rhFGF20 across the blood–brain barrier (BBB) have halted its clinical application. In the present study, we have examined the efficiency of fuzing a small ubiquitin-related modifier (SUMO) to rhFGF20 to enhance its soluble expression and further investigated the efficacy of FUS-guided, rhFGF20-liposome transport across the BBB. We also examined the bioavailability and behavioral improvement in a 6-hydroxydopamine-lesioned rat model of PD following 2 weeks’ FUS-liposomal combinatorial treatment. Our results showed that, in contrast with rhFGF20 or LIP-FGF20, the FUS-LIP-rhFGF20 treatment could significantly improve the apomorphine-induced rotations by protecting against the loss of dopaminergic neurons in the SNpc. Our Results suggest that our combinatorial method would help overcome key challenges that hinder the currently available methods for the use of rhFGF20 in PD treatment.

## Introduction

Parkinson’s disease (PD) is a neurodegenerative disorder that affects 1–2 per 1000 populations globally. The major pathological feature in PD is the loss of dopaminergic neurons (DA) located in the substantia nigra (SN) of the midbrain (Hartmann et al., 2000). Fibroblast growth factor-20 (FGF20) is a paracrine member of the FGF family of growth factors (Beenken & Mohammadi, 2009) that is predominantly expressed in the mid-brain, especially the substantia nigra pars compacta (SNpc) (Ohmachi et al., 2000; Thuret et al., 2004). This expression profile has prompted researchers to explore the relationship between FGF20 and PD (Ohmachi et al., 2003; Correia, 2007; Itoh & Ohta, 2013). In fact, accumulating evidences upon its well-known neurotrophic activity support an essential role of FGF20 in enhancing the development and survival of dopaminergic neurons *in vitro* (Grothe et al., 2004; Correia, 2007; Shimada et al., 2009). Exogenous recombinant human FGF20 (rhFGF20) has been previously shown to promote the differentiation of human embryonic stem cells into dopaminergic neurons and attenuate the 1-methyl-4-phenyl-1,2,3,6-tetrahydropyridine (MPTP)-induced neurological symptoms (Shimada et al., 2009). Further, FGF20 has been shown to provide functional protection against the loss of dopaminergic neurons in a 6-hydroxydopamine-lesioned rat PD model (Sleeman et al., 2012). The neurotrophic activity of FGF20 is mediated by activation of receptor FGFR1c, which in turn triggers the activation of the MAPK cascade and phospholipase C-γ pathway (Ohmachi et al., 2003; Thisse & Thisse, 2005). Additionally, FGF20 polymorphism (rs1721100(C/G)) was identified as a risk factor for PD in Chinese Han population (Pan et al., 2012).

To date, rhFGF20 is heterologously expressed in *Escherichia coli* system, wherein the high production yield of recombinant rhFGF20 obtained often leads to misfolded and aggregated FGF20 proteins resulting as insoluble, inclusion bodies that need time-consuming *in vitro* protein refolding methods that lead to protein loss (Basu et al., 2011). Additionally, the blood–brain barrier (BBB) blocks large molecules (>400 Da) from penetrating the brain parenchyma (Patel & Patel, 2017). Therefore, the expression of rhFGF20 in the form of inclusion bodies and the absence of an efficient delivery approach into the brain hinder both the basic research and clinical application of rhFGF20 in PD treatment.

Over many years, several strategies have been developed to enhance the solubility of recombinant proteins including reduction of the culture temperature, lowering of inducer concentrations and the use of fusion tags (Middelberg, 2002). Of these, small ubiquitin-related modifier (SUMO) proteins have gained much attention as an effective fusion system that enhances the expression of soluble recombinant proteins (Liu et al., 2012). More recently, the technology of focused ultrasound (FUS) technology that can reversibly open the BBB in a site-specific manner has been experimentally established as a noninvasive and localized brain drug delivery technique (Park et al., 2012; Aryal et al., 2014; Aryal et al., 2015). Neuroprotective agents and antibodies were successfully transported across the BBB following FUS-induced BBB disruption (Kobus et al., 2016). In addition, biodegradable nanoparticles (NP) can protect the proteins and genes from destruction by *in vitro* and *in vivo* factors (Tian et al., 2017). Therefore, highly efficient and safe delivery systems can be devised/developed using a combination of FUS and biodegradable NP.

In the present study, we firstly aimed to achieve soluble expression of rhFGF20 by introducing the SUMO tag to the N-termini of rhFGF20. The second purpose of this study was to determine whether a combination therapy of FUS directed rhFGF20-loaded liposomes was effective in providing functional neuroprotection in a rat PD model. To achieve an in-depth understanding of the therapeutic impact of the FUS + LIP-rhFGF20 technique, a broad range of commonly used pathophysiological indicators were measured in a PD rat model. These measurements allowed for a thorough preclinical evaluation of the *in vivo* effects of FUS + LIP-rhFGF20 treatment on the neuroprotection functions and related structural damages following two weeks’ treatment. Overall, this combination therapy promises to be an efficient adjuvant therapy to cure pathophysiology of neurodegenerative disorder in PD patients.

## Material and methods

### Experimental materials

BL21 (DE3) *E. coli* stain was purchased from Novagen Inc (Madison, WI). Methylthiazol tetrazolium (MTT) and IPTG were purchased from Bio-Tech (Gold Biotechnology, Inc., Olivette, MO). Dulbecco’s modified Eagle’s medium (DMEM) and fetal bovine serum (FBS) were purchased from Invitrogen (Carlsbad, CA). PC-12, NIH 3T3 and SH-SY5Y cell lines were purchased from the Shanghai Institute of Cell Biology Institute library, Chinese Academy of Sciences (Shanghai, China). Rabbit anti-human FGF20 polyclonal antibody, rabbit anti-rat caspase-3 polyclonal antibody and rabbit anti-rat tyrosine hydroxylase (TH) polyclonal antibody were purchased from Cell Signaling Technology (Danvers, MA).

### Expression and purification of SUMO-rhFGF20

Competent BL21 (DE3) *E. coli* cells were transformed with pET20b-SUMO-rhFGF20 which was obtained as previously reported (Wang et al., 2010), and were cultured at 37 °C to an A_600_ of 0.8 (Beckman DU530 UV–visible spectrometer) and induced with 1 mM isopropyl-l-thio-b-d-galactopyranoside (IPTG) at 20 °C for 16 h. The degree of protein expression was evaluated by 10% sodium dodecyl sulfate–polyacrylamide gel electrophoresis (SDS–PAGE). Next, the culture mixture was centrifuged at 4000*g* for 5 min; the pellet was resuspended in lysis buffer [20 mM Tris–HCl, pH 8.0, 10% (V/V) glycerol and 150 mM NaCl] and disrupted by intermittent sonication in an ice bath. The soluble fraction and insoluble pellet were subjected to SDS-PAGE analysis. Ni-affinity chromatographic purification were performed using Ni-affinity chromatography [column volume (CV)=10 ml] as detailed previously (Wang et al., 2010).

### Cleavage of SUMO from SUMO-rhFGF20 and purification of rhFGF20

The cleavage of SUMO from SUMO-rhFGF20 was performed as previously described (Wang et al., 2010). Briefly, 1 mg/ml SUMO-rhFGF20 and 50 U of SUMO protease were mixed and incubated at 4 °C for 16 h. Next, the mixture was loaded onto a Source Q column. The SUMO, SUMO-rhFGF20 as well as SUMO protease were eluted with 10 volumes of Buffer A (25 mM Tris–HCl, 0.8 M NaCl) and rhFGF20 was eluted by Buffer B (25 mM Tris–HCl, 1.5 M NaCl) according to the manufacturer’s protocol. Different elution fractions were collected and analyzed by 10% SDS–PAGE and western blotting using a monoclonal mouse anti-hFGF20.

### Biological activity of rhFGF20 on NIH3T3, PC-12 and SY-SY5Y cells

For *in vitro* bioactivity, NIH 3T3 cells were used to determine the activity of rhFGF20 compared to FGF2 as a positive control (Zhu et al., 2015). Cells were seeded in a 96-well tissue culture plates (1.0 × 10^4^ cells/well) with DMEM supplemented with 10% (v/v) FBS. Then, cells were serum-starved for 24 h followed by treatment with various concentrations of rhFGF20 and FGF2 for another 24 h. The proliferation rate was determined with the MTT assay as reported previously (Tian et al., 2016).

In this study, we also investigated the neuroprotective effect of rhFGF20 using a six-hydroxydopamine (6-OHDA)-induced apoptosis on PC-12 and SH-SY5Y cells. Briefly, PC-12 and SH-SY5Y cells were seeded in a 96-well microplate (1.0 × 10^3^–1.0 × 10^4^ cells/well) with DMEM supplemented with 10% (v/v) FBS. The cells were serum starved for 24 h and treated with various concentrations of 6-OHDA for another 24 h to investigate its neurotoxicity. Following serum starvation, PC-12 and SH-SY5Y cells were incubated with 120 μM and 100 μM 6-OHDA, respectively for 24 h; then different concentrations of rhFGF20 and FGF2 were added for another 24 h. MTT proliferation assays were performed as previously reported (Tian et al., 2016).

Finally, we examined whether the protective effect of rhFGF20 against 6-OHDA was mediated via inhibiting apoptosis. To this end, treated PC12 cells were lysed in Protein Extraction Buffer (Sigma-Aldrich, St. Louis, MO) with protease and phosphatase inhibitors and subjected to western blotting to determine the levels of caspase-3. The second part was used immunofluorescence staining to observe the changes of caspase-3 expression. The rest part was carried on terminal deoxynucleotidyl transferase-mediated deoxyuridine triphosphate nick end labeling (TUNEL) staining. The apoptotic cells were detected by the In Situ Cell Death Detection Kit (Roche Molecular Biochemicals, Mannheim, Germany) according to the manufacturer’s instructions. The images were visualized using a fluorescence microscope (Olympus, Tokyo, Japan). To quantitatively examine the numbers of apoptotic cells, the TUNEL-positive cells were counted.

### Immunofluorescence staining

Immunofluorescence assays were used to assess the protein levels of cleaved caspase-3. The PC12 cells were planted on glass coverslips in six-well plates. After treatment, samples were fixed with 4% PFA for 1 h, and permeabilised by 0.5% Triton X-100 for 10 min. After block with 1% bovine serum albumin (BSA) at 4 °C for 45 min, the primary antibodies were incubated overnight. The next day, samples were incubated with Alexa Fluor 488-conjugated anti-IgG secondary antibodies for 1 h and stained with DAPI for 7 min. Samples were observed by a Leica TCS SP8 microscope (Berlin, Germany).

### Preparation and characterization of rhFGF20-loaded liposomes

RhFGF20-loaded liposome (LIP-rhFGF20) was prepared by reverse-phase evaporation. Briefly, the phospholipid membrane was completely dissolved in 10 ml dichloromethane at room temperature. Then, rhFGF20 solution was added into a dichloromethane solution drop wise, followed by an ultrasonic probe ultrasound until a W/O emulsion was formed. Later, vacuum evaporation was performed in order to remove dichloromethane. The resulting liposomes were prepared in the desired protein concentration and stored at −80 °C. The morphology of LIP-rhFGF20 was confirmed by transmission electron microscopy (TEM). The particle size distribution of LIP-rhFGF20 was determined by dynamic light scattering as described previously (Tian et al., 2016).

The *in vitro* release of rhFGF20 from LIP-FGF20 was evaluated as previously reported (Sun et al., 2014). Briefly, 1 mL of the prepared LIP-FGF20 solution (equivalent to 2.5 mg/mL rhFGF20) or free rhFGF20 solution (2.5 mg/mL rhFGF20) were placed in the dialysis membrane (MWCO = 50 kDa) and dialyzed against 9 mL PBS containing 0.5% Tween-80 as an external solution for dialysis. Dialysis was performed on a magnetic stirrer at 37 °C. The rhFGF20 concentrations in the external solution at different time points were determined by a human FGF20 ELISA Kit (Abbexa Ltd., Cambridge Science Park, Cambridge, UK). The cumulative release percentage (%) was determined by dividing the cumulative amount of free rhFGF20 recovered in the release medium at each time point by the total amount of rhFGF20 in 1 mL of LIP-rhFGF20 solution.

### The in vivo neuroprotective effect of FUS + LIP-rhFGF20

#### Establishment of PD rat model

Adult male Sprague-Dawley rats (280–300 g) were purchased from the Model Animal Research Center of Nanjing University, China, and the protocols used in these studies were approved by the Animal Care and Use Committee of Wenzhou Medical University, China. Animals were housed in a specific pathogen-free animal facility with controlled environment (22±°C, 50–60% humidity, 12-hour light–dark cycle, lights on at 7 am) and free access to food and water. A total of 40 rats were divided into two groups, one group receiving 6-OHDA treatment (6-OHDA rats; total =30) and the other group receiving normal saline treatment (sham rats; *n* = 10). Rats were anesthetized with an intraperitoneal (i.p.) injection of chloral hydrate (350 g/kg body weight) and placed into a stereotaxic apparatus (AP +1.0 mm, ML +3.0 mm, and DV +4.5 mm). Next, rats were administered either 6-OHDA (10 μl at 16 μg/μl, dissolved in 0.1% ascorbic acid-saline) or normal saline into the right striatum (AP +1.0 mm, ML +3.0 mm, and DV +4.5 mm) (Zhu et al., 2015). The injection rate was controlled by an auto-injector attached to the stereotaxic apparatus at a rate of 1 μl/min. Next, rats were examined for rotational behavior induced by apomorphine hydrochloride injection (0.5 mg/kg; i.p) at 3 and 4 weeks following 6-OHDA injection. Rats exhibiting significantly high contralateral rotational rate (>7 turns/min) were identified as a valid PD model and subjected to further studies (Zhu et al., 2015).

#### RhFGF20 treatment and rotational behavior test

PD-induced rats were randomly subdivided into four groups: normal saline-treated group (control group; *n* = 6), rhFGF20-treated group (*n* = 6), LIP-rhFGF20 treated group (*n* = 6) and FUS + LIP-rhFGF20 treated group (*n* = 6). Normal saline or different proteins were injected once daily for 2 weeks into the tail vein, all the dosages of rhFGF20, LIP-rhFGF20 and FUS + LIP-rhFGF20 group were 0.5 mg/kg. The FUS effect used in this study was generated by a linear array transducer (Acuson Sequoia 512C System; Siemens, Berlin, Germany). The parameters of the transducer sonication in the FUS + LIP-rhFGF20 group were set as follows: ultrasound frequency 0.69 MHz, pulse repetition frequency of 1 Hz, sonication time of 60 s and burst length of 10 ms with an acoustic power 3 W. The rotation behavior assay was repeated after 2 weeks as mentioned above (Zhu et al., 2015).

### Measurement of monoamine neurotransmitters and their metabolites in the striatum

The concentrations of monoamine neurotransmitters and their metabolites in the striatum were determined by HPLC-electrochemical detection (ECD) as we reported previously (Zhu et al., 2015). Briefly, rats were anesthetized with 4% choral hydrate, decapitated and the striatum samples were sonicated and homogenized in chilled 0.1 M HClO_4_ (1 mL/200 mg tissue). Following centrifugation (10,000 g; 15 min), the supernatant was collected and diluted with the mobile phase (75 mM NaH_2_PO_4_, 1.7 mM octane sulfonic acid and 10% methanol, pH 3.9). A 10 μL sample was isocratically eluted through a 150 × 3.5 mm C18-column (Agilent Technologies Inc., Lexington, MA) at a flow rate of 1.0 mL/min. Neurotransmitters and metabolites including dopamine (DA), 3,4-dihydroxyphenylacetic acid (DOPAC), and homovanilic acid (HVA) were detected by a two-channel electrochemical detector (Waters & Associates, St. Louis, MO) at a potential of 1.5 mV. Concentrations were normalized to wet tissue weight.

#### Histology and immunohistochemistry

Rats were anesthetized with 4% choral hydrate (10 ml/kg; i.p) and perfused with 4% paraformaldehyde in a 0.1 M phosphate buffer. Brains were dissected and post-fixed in the same fixative overnight at 4 °C. The substantia nigra were dehydrated in an ascending ethanol series, embedded in paraffin and sectioned into 5–7 μm sections using a microtome (Leica). Following tissue rehydration, dewaxing and antigen retrieval at high temperature and pressure, sections were incubated with 3% hydrogen peroxide for 30 min and 5% BSA for 30 min at 37 °C. Subsequently, sections were incubated with primary rabbit anti-TH antibody (1:5000) at 4 °C overnight. Next, sections were washed with PBS and incubated with biotinylated secondary anti-rabbit antibody for 1 h at 37 °C, followed by incubation with streptavidin-HRP conjugate. Treated sections were incubated with 3,3-diaminobenzidine (DAB, 5% diluted with PBS) for 1 min. Sections obtained from the sham group served as a negative control. The number of positive cells from each group were counted at ×400 using a Nikon ECLPSE 80i (Nikon, Tokyo, Japan) and the difference between groups was analyzed.

#### Western blotting

All protein samples were separated on a 12% gradient SDS–PAGE and transferred to PVDF membranes (Bio-Rad, Hercules, CA). Membranes were blocked in 10% skim milk for 1 h, and incubated with primary anti-TH antibody (1:5000) (Cell signaling Technology, Danvers, MA) or GAPDH (1:5000) (Cell signaling Technology, Danvers, MA) overnight at 4 °C. Then, membranes were washed with Triton X-100 in TBS 3 and incubated for 1 h with appropriate HRP-conjugated secondary antibodies at room temperature. Blots were washed and exposed using a ChemiDoc™ XRS + Imaging System (Bio-Rad, Hercules, CA). Densitometry analysis band intensity was performed using Multi Gauge Software, Science Lab 2006 (FUJIFILM Corporation, Tokyo, Japan).

### Determination of exogenous rhFGF-20 in the striata

Striatal tissues from different groups were homogenized in lysis buffer as previous report (Zhu et al., 2015) and centrifuged at 12,000*g* and 4 °C for 15 min in order to determine the concentration of rhFGF-20 after treatment with LIP-rhFGFA20 or FUS + LIP-rhFGFA20. The concentration of rhFGF20 in the supernatant were measured by a human fibroblast growth factor-20 (hFGF20) ELISA Kit (Antibodies, UK) according to the manufacturer’s protocol. Total protein concentration was determined by bicinchoninic acid (BCA) Protein Assay Kit (Thermo Scientific, Waltham, MA). Concentrations of rhFGF20 were normalized to the total protein concentration in each sample.

### Statistical analysis

All data were presented as the mean ± standard error of mean (SEM). Multiple groups were tested using one-way analysis of variance (ANOVA) followed by Dunnett’s test to determine the statistically significant groups, using Origin 7.5 (OriginLab, Northampton, MA) for data analysis. A *p* value <.05 (*) was considered to be statistically significant.

## Results and discussion

### Expression and purification of rhFGF20

As a member of the FGF ligands paracrine subfamily, FGF20 plays an important role in tissue formation and regeneration, with well-known neuroprotective functions in neurodegenerative diseases (Itoh & Ohta, 2013; Tian et al., 2016). For instance, Sleeman et al. (Sleeman et al., 2012), previously reported that the chronic supra-nigral infusion of FGF20 protected against the loss of TH-positive cells in the SNpc and the loss of striatal TH immunoreactivity in PD rat model. These reports suggest a potential neuroprotective role for FGF20 in PD. However, obtaining biologically active, recombinant FGF20 from inclusion bodies by *in vitro* protein refolding methods is a complicated and time-consuming process, which hinders the wide clinical use of efficacious FGF20.

**Figure 1.**
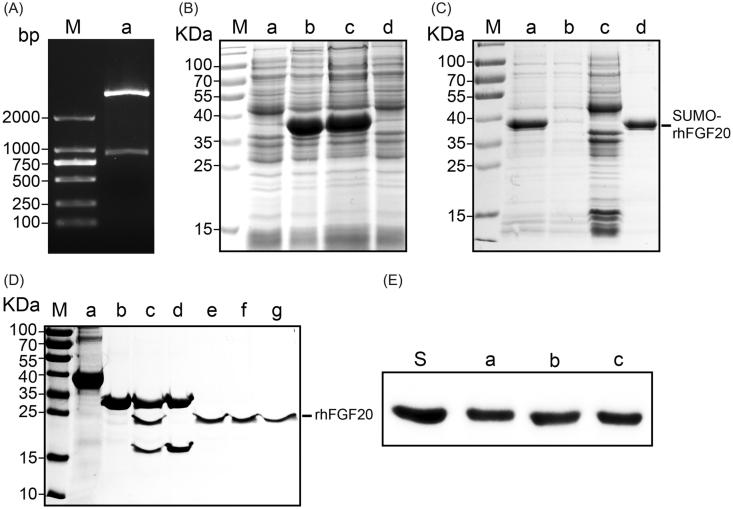
Soluble expression and purification of rhFGF20. (A) Agarose gel electrophoresis showing the molecular weight of the insert DNA comprising the SUMO-rhFGF20 DNA sequence (lane a, lower band ∼1000 bp) and the backbone of pET20 expression vector (lane a, upper band >2000 bp). (B) SDS–PAGE gel showing expression of SUMO-rhFGF20. Lane M: protein molecular weight markers; lane a: bacterial lysate before IPTG induction; lane b: bacterial lysate after induction by 1 mM IPTG; lane c: soluble supernatant fraction after centrifugation; lane d: insoluble fraction of the bacterial lysate after low-speed centrifugation. (C) SDS–PAGE gel analysis for fractions from Ni–NTA agarose column. Lane M: protein molecular weight markers; lane a: loading sample; lane b: flow through; lane c: wash fraction; lane d: fraction eluted from Ni column. (D) Source Q purification following digestion by SUMO protease. Lane M: protein molecular weight markers; lane a: IMAC purified SUMO-rhFGF20; lane b: SUMO protease; lane c: sample after enzyme digestion; lane d: SUMO protease and SUMO eluted from Source Q column; lane e–g: purified rhFGF20. (E) Immunoblotting of the purified rhFGF20 detected using anti-human FGF20 antibody. Lane S: standard rhFGF20 sample; Lane e–g: purified rhFGF20.

The SUMO fusion system was demonstrated to enhance the soluble expression of various recombinant proteins including FGF21, FGF23, insulin-like growth factor-1 (IGF-1) (Wang et al., 2010; Sun et al., 2014; Tian et al., 2016). In the present study, we hence aimed to obtain soluble expression of bioactive rhFGF20 using the SUMO fusion tag. The pET20 expression vector harboring human FGF20 (633 bp) and a 318 bp SUMO tag was transformed into BL21 (DE3) *E. coli* cells (Figure 1(A)), and the expression of proteins was induced by 1 mM IPTG. The soluble and pellet fractions of the cell lysate were analyzed on a 12% (v/v) SDS–PAGE alongside other protein purification fractions (Figure 1(B)). Ni-affinity chromatography was used for the purification of SUMO-rhFGF20. And the proteins were eluted at 400 mM imidazole in 25 mM Tris–HCl buffer (pH = 8.0,150 Mm NaCl). Recombinant SUMO-rhFGF20 was identified between 35 and 40 kDa bands that corresponds to the predicted molecular mass of SUMO-rhFGF20 (Figure 1(C)).

As described in previous reports, the pre-purified SUMO-rhFGF20 was diluted and the C-terminal SUMO tag was cleaved by SUMO protease at 4 °C for 12 h (Wang et al., 2010). Anion exchange column, Source Q was used as the final polishing step in the purification of FGF20 where we obtained pure, SUMO tag cleaved rhFGF20 (24 kDa) (Figure 1(D)). The identification of rhFGF20 was confirmed by Western blotting using human FGF20 polyclonal antibody (Figure 1(E)).

### The in vitro bioactivity assay of rhFGF20

**Figure 2.**
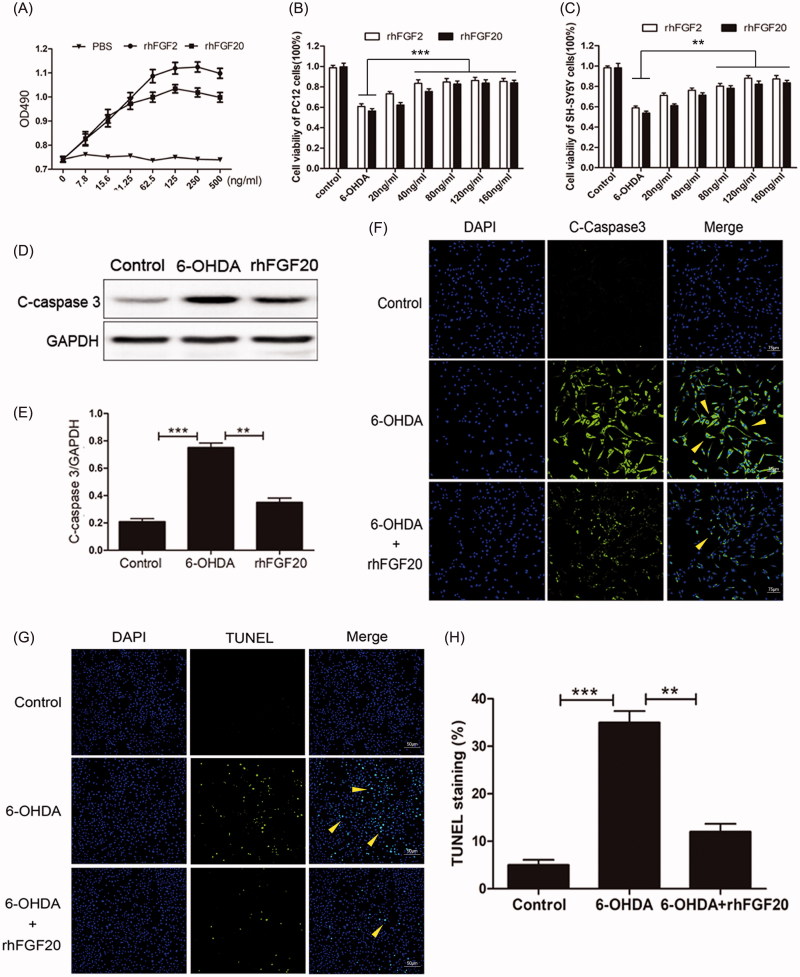
Activity assay of purified rhFGF20. (A) Mitogenic activity of rhFGF20 and rhFGF2 in NIH 3T3 cells. Purified rhFGF20-induced mitogenic response in NIH3T3 cells in a dose-dependent manner that was comparable to that of rhFGF2 with the curve saturating at 125 ng/ml. (B,C) Comparison of the effects of FGF20 and FGF2 on the viability of PC12 (B) and SHSY5Y (C) cells respectively. (D,E) Western blotting and semi-quantitative analysis of caspase-3 levels in PC-12 cell induced with 6-OHDA or 6-OHDA/rhFGF20. (F) Immunofluorescence staining of caspase-3 levels in PC-12 cell induced with 6-OHDA or 6-OHDA/rhFGF20. Scale bar = 75 μm. (G,H) Representative pictures of the TUNEL staining (apoptotic cells are indicated by yellow arrows). Quantitative analysis of the apoptotic cells. Scale bar = 50 μm. Data from three independent measurements are presented as mean ± SEM. **p < .01; ***p < .001 versus 6-OHDA group.

In order to evaluate the *in vitro* activity of rhFGF20, we performed the MTT assay to analyze the proliferative potential of rhFGF20 on NIH-3T3. Additionally, since FGF2 has been previously shown to enhance the proliferation of the fibroblast cell line NIH-3T3 in a dose-dependent manner (Jia et al., 2015; Zhu et al., 2015), we determined the proliferative potential of rhFGF20 using FGF2 as a positive control. We observed that purified rhFGF20 induced a mitogenic response in NIH3T3 cells in a dose-dependent manner, comparable to that of FGF2 (Figure 2(A)), these values are consistent with previously reported activity of rhFGF20 purified from inclusion bodies by protein refolding methods (Tian et al., 2016).

6-OHDA, a hydroxylated analog of dopamine, induces pronounced oxidative stress in DA neurons (Schober, 2004). Therefore, 6-OHDA-induced apoptosis is considered to be a well-established *in vitro* model for PD (Walkinshaw & Waters, 1994). The rat PC-12 cell line and the human SH-SY5Y cell line can be differentiated into neuronal-like cells and they can be used as an i*n vitro* model for PD (Walkinshaw & Waters, 1994). We also assessed the anti-apoptotic activity of rhFGF20 in an *in vitro* PD model the latter established by treating PC-12 and SH-SY5Y cells with 125 μM 6-OHDA for 24 h. Following this, cells were stimulated with different concentrations of rhFGF20. Our results demonstrate that that the survival rates of PC-12 and SH-SY5Y cells treated with 125 μM 6-OHDA were decreased by 39 and 41%, respectively. However, following stimulation with different concentrations of rhFGF20, the survival rates of the two types of cells could be improved to ∼83 and 84%, respectively. Hence, our MTT assay confirmed that purified rhFGF20 protected PC-12 and SH-SY5Y cells against 6-OHDA-induced apoptosis in a dose-dependent manner (20 and 160 ng/mL) (Figure 2(B,C)).

PD is characterized by the progressive loss of DA neurons due to apoptotic cell death (Tatton et al., 2003). In order to investigate whether rhFGF20 exerted its neuroprotective effect to revive 6-OHDA-induced apoptosis in PC-12 cells by modulating the anti-apoptotic pathway, we examined the expression of caspase-3 by western blotting. And indeed, the level of caspase-3 was up-regulated in PC-12 cells treated with 6-OHDA compared to vehicle treated cells and this increase was reduced by co-treatment with rhFGF20 (Figure 2(D,E)), accompanied by the caspase-3 (Figure 2(F)) and TUNEL (Figure 2(G,H)) immunofluorescence staining results which demonstrated that the PC-12 cells apoptosis induced by 6-OHDA could be ameliorated by FGF20 treatment. These results indicate that the neuroprotective activity of rhFGF20 is partly mediated by modulating the anti-apoptotic activity.

### Characterization and in vitro-release of LIP-rhFGF20

**Figure 3.**
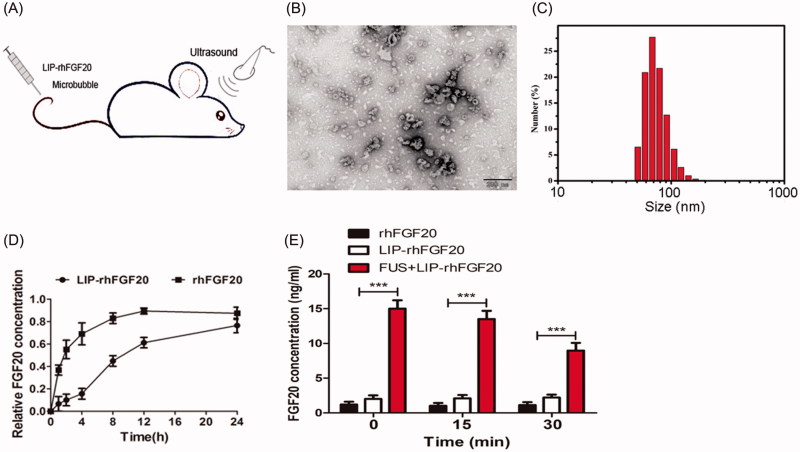
Characterization of LIP-rhFGF20. (A) Dosing diagram for FUS + LIP-rhFGF20. Normal saline or different proteins were injected once daily for 2 weeks into the tail vein. For FUS + LIP-rhFGF20 treatment, for each administration of LIP-rhFGF20, ultrasound was focused on the appropriate part of the brain of the mouse. (B) Morphology of LIP-rhFGF20 as analyzed by scanning electron microscopy (JEOLJEM-2000EX; JEOL, Tokyo, Japan) at 200 kV. (C) The particle size and distribution of LIP-rhFGF20 showing uniform distribution of 68.1 ± 2.1 nm (PDI = 0.144). (D) The *in vitro* release of rhFGF20 from LIP-rhFGF20 over a 24 h time period, showing purified rhFGF20 as control sample. (E) Determination of concentration of exogenous rhFGF-20 in the striata of 6-OHDA rats. The rhFGF-20 concentrations in striatal tissue of PD rats treated with FUS + LIP-rhFGF20, LIP-rhFGF20 and rhFGF20 were measured at 0, 15 and 30 min following treatment. Data from three independent measurements are presented as mean ± SEM. ***p < .001 versus rhFGF20 or LIP-rhFGF20.

Overcoming the selective permeability of the BBB increases the odds of directed delivery of therapeutic agents for multiple brain disorders, including PD, Alzheimer’s disease, stroke and brain injury (Patel & Patel, 2017). Nevertheless, invasive drug delivery approaches are hampered by possible side effects like the penetration of cytotoxic drugs into non-targeted areas (O’Reilly et al., 2011). Recent reports showed that, FUS combined with circulating micro-bubbles offered a reliable method to disrupt the BBB in a targeted, noninvasive and reproducible manner (Tian et al., 2016; Vlachos et al., 2010), enhancing drug delivery into the brain. However, the poor stability of the therapeutic agents usually affects their long-term efficacy. Therefore, in this study, we combined FUS with LIP-rhFGF20 to open the BBB in a noninvasive manner with prolonged *in vivo* half-life of rhFGF20 for targeted neuronal therapy (Figure 3(A)). Transmittance electron microscopy of LIP-rhFGF20 demonstrated a spherical to slightly elliptical shape of the LIP particle (Figure 3(B)). Dynamic light scattering experiments exhibited an average diameter of 68.1 ± 2.1 nm and a low polydispersity index (PDI) (0.144) for the LIP-rhFGF20, indicating that these liposomes had a narrow size distribution ideal for targeted LIP therapy (Figure 3(C)). These structural parameters promised to enable the transportation of LIP-rhFGF20 across the BBB.

Further, we examined the *in vitro* drug release rate to evaluate the release rate of free rhFGF20 from LIP-rhFGF20 using purified rhFGF20 protein as control. We observed that the release rate of LIP-rhFGF20 was significantly lower than that of pure rhFGF20 (Figure 3(D)). At 2-h post-dialysis experiment, rhFGF20 solution released 50% free rhFGF20, whereas LIP-rhFGF20 released only 15% free rhFGF20 (Figure 3(D)). Therefore, LIP-rhFGF20 system could be applied for sustained rhFGF20 release, exerting long-lasting effects as an efficacious therapeutic agent.

In order to verify that FUS temporarily opens the BBB and that the exogenous rhFGF20 enters the brain, we evaluated the levels of rhFGF20 in the rat striatum at different time points following treatment with rhFGF20, LIP-rhFGF20 or FUS + LIP-rhFGF20. Indeed, the level of rhFGF20 in the FUS + LIP-rhFGF20 group was significantly enhanced compared with those of LIP + rhFGF20 or rhFGF20 group at all the examined time points (Figure 3(E)). This result testified that the bioavailability of the FUS + LIP-rhFGF20 in the brains of 6-OHDA rats was significantly enhanced compared to both rhFGF-20 alone or LIP-rhFGF20.

### Combination of LIP-rhFGF20 and FUS ameliorated the 6-OHDA-induced neurobehavioral deficit

**Figure 4.**
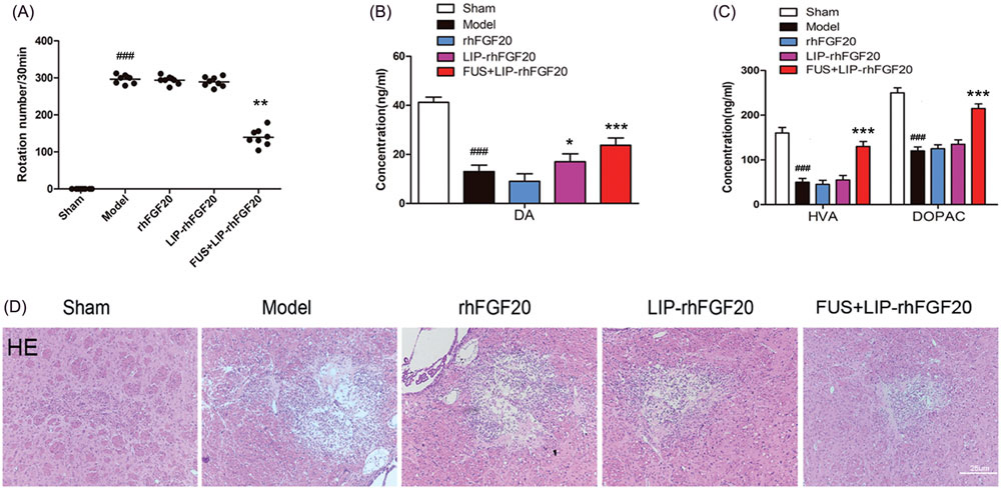
Effects of FUS + LIP-rhFGF20 on amphetamine-induced rotational behavior, and concentrations of monoamine neurotransmitters and their metabolites in the striatum of 6-OHDA-induced rat model of PD. (A) Effects of rhFGF20, LIP-rhFGF20, and FUS + LIP-rhFGF20 on apomorphine-induced rotational behavior: DA (B) and HVA/DOPAC (C) concentrations in striatum tissue were determined by HPLC-ECD. (D) Detection of tissue damage in striatum by HE staining after 2 weeks’ treatment. Scale bar =25 μm. Data are presented as mean ± SEM (*n* = 6). *p < .05; **p < .01; ***p < .001 versus 6-OHDA group; ###*p* < .001 versus sham rats.

We performed *in vivo* studies to evaluate the therapeutic effects of FUS + LIP-rhFGF20 in 6-OHDA-induced PD rat model by determining behavioral defects, neurotransmitter levels and tissue damage. Apomorphine-induced rotational behavior showed that, the combination of LIP-FGF20 and FUS significantly reduced the apomorphine-induced contralateral rotation in 6-OHDA rats by 50%, whereas treatment with rhFGF20 and LIP-rhFGF20 did not led to any reduction after 2 weeks’ treatment interval (Figure 4(A)). These results demonstrated that FUS + LIP-rhFGF20 enhanced the efficacy of rhFGF20 in ameliorating 6-OHDA-induced neurobehavioral deficit. In the meantime, a control group treated by ultrasound treatment without any therapeutics has been added to examine the histological change. The result clearly showed that ultrasound treatment didn’t cause any extravasation of red blood cells (Supplementary Figure S1), indicating the safety of this method. This result is consistent with previous studies (Konofagou, 2012; O’Reilly et al., 2011; Vlachos et al., 2010).

Behavioral improvement in neurological disorders is probably associated with changes in monoamine neurotransmitters and their metabolites including DA, HVA and DOPAC (Zhu et al., 2015). Hence, we determined the impact of FUS + LIP-rhFGF20, LIP-rhFGF20 and rhFGF20 on the levels of DA, HVA, and DOPAC in the lesioned striata. Compared to the sham group, the concentrations of DA, HVA, and DOPAC in the 6-OHDA group were reduced to 27, 29, and 54%, respectively, (Figure 4(B,C)). Treatment with rhFGF20 and LIP-rhFGF20 showed no significantly difference with the 6-OHDA group, whereas, treatment with FUS + LIP-rhFGF20 significantly alleviated the 6-OHDA-induced decrease in DA, HVA, and DOPAC levels (Figure 4(B,C)). Histology examination by the hematoxylin–eosin staining showed that improvements of behavioral defects and neurotransmitter levels were due to that FUS + LIP-rhFGF20 could significantly alleviate the tissue damage induced by 6-OHDA in the striatum, however, neither rhFGF20 alone or as LIP-rhFGF20 showed any significant effect on this damage (Figure 4(D)).

### Combination of LIP-rhFGF20 with FUS-alleviated 6-OHDA-induced dopaminergic neural loss

**Figure 5.**
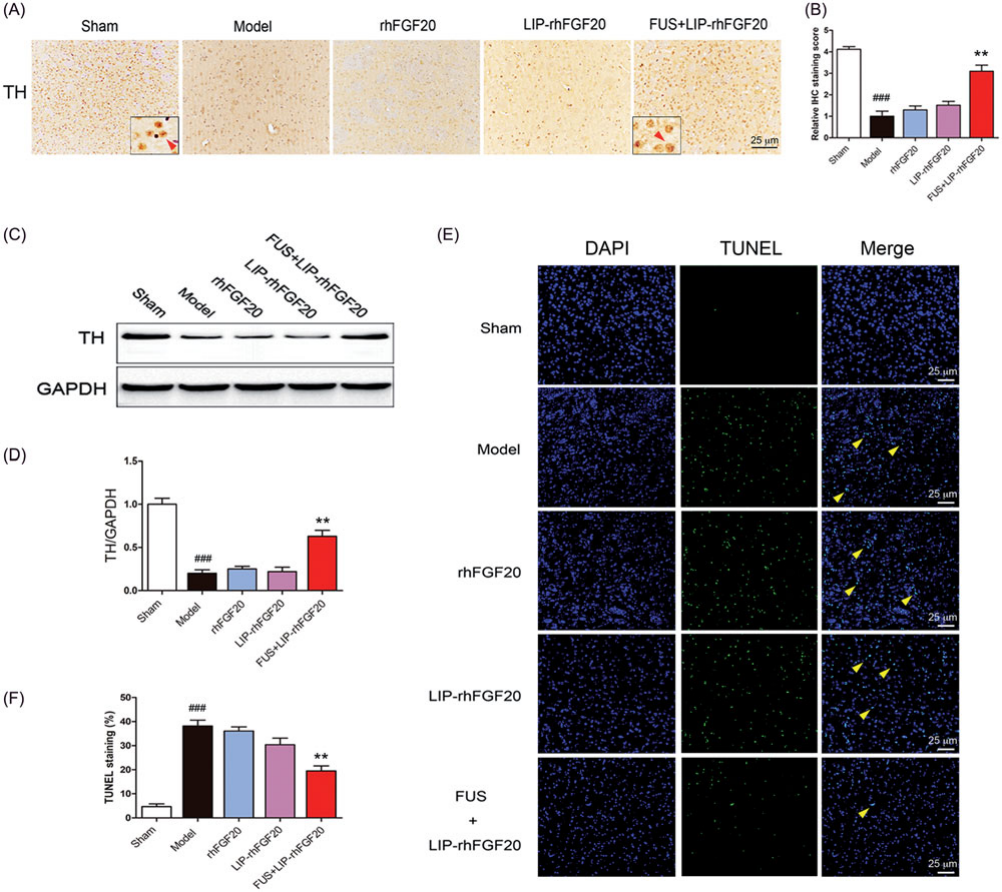
Impact of FUS + LIP-rhFGF20 on the dopaminergic neural loss in the striatum of 6-OHDA rats. (A) Immunohistochemistry assay using TH-antibody were carried out to detect the effects of FUS + LIP-rhFGF20, LIP-rhFGF20 and rhFGF20 on the levels of TH-positive dopaminergic neurons in SN tissues. Scale bar =25 µm. (B) Semi-quantitative analysis of TH immunohistochemistry observed in panel A. (C) Effect of FUS + LIP-rhFGF20, LIP-rhFGF20 and rhFGF20 treatment on the protein levels of TH in SN tissues. (D) Semi-quantitative analysis of the protein bands observed in panel C. (E,F) After 2 weeks’ treatment, analysis of the striatum tissue by TUNEL staining was performed to assess the effects of drugs on apoptosis. Representative pictures of the TUNEL staining (apoptotic cells are indicated by yellow arrows). Quantitative analysis of the apoptotic cells. Scale bar = 25 μm. All data in this figure from three independent measurements are presented as mean ± SEM. **p < .01 versus 6-OHDA group; ###*p* < .001 versus sham rats.

Behavioral improvement can be caused by increased dopaminergic neural survival (Tolleson & Fang, 2013). Therefore, we evaluated the survival of dopaminergic neurons via immunostaining for TH-positive neurons within the SN stain and immunoblot analysis for TH protein. Injection of 6-OHDA toxin resulted in a marked loss of TH-positive dopaminergic neurons in the lessoned side of SN (Figure 5(A,B)) and a concurrent reduction in TH protein expression (Figure 5(C,D)). Because it can’t penetrate the BBB very well, a 2-week treatment with either rhFGF20 or LIP-rhFGF20 was not capable of preventing the loss of TH-positive neurons in the SN of 6-OHDA rats (Figure 5(A,B)). Notably, the number of TH-positive neurons in the FUS + LIP-rhFGF20 treatment group almost approached that of the levels of sham group (Figure 5(A,B)). Consistent with the IHS data, treatment with FUS + LIP-rhFGF20 led to a greater increase in TH protein expression than treatment with either rhFGF20 or LIP-rhFGF20 (Figure 5(C,D)). As shown in cell-based experiments, we also noticed that the SN apoptosis induced by 6-OHDA could be ameliorated by a two-week FUS + LIP-rhFGF20 treatment (Figure 5(E,F)), hence confirming that FUS + LIP-rhFGF20 exhibited greater efficacy in protecting the cells against 6-OHDA-induced neurotransmitter deficiency compared to either rhFGF20 or LIP-rhFGF20 alone.

## Conclusions

In this study, we achieved an enhanced solubility of rhFGF20 by introducing a SUMO tag. Moreover, we showed that rhFGF20 could be delivered efficiently into the brain by using FUS-guided rhFGF20 liposomes, which significantly improved the apomorphine-induced rotations by protecting against the SNpc dopaminergic neural loss. To the best of our knowledge, this is the first study that achieves enhanced solubility, delivery and bioavailability of rhFGF20. Results obtained from this study will in instrumental in overcoming key challenges that hinders the basic research and clinical application of FGF20 in the treatment of PD.

## Supplementary Material

Supplementary Figure S1

## References

[CIT0001] Aryal M, Arvanitis CD, Alexander PM, et al (2014). Ultrasound-mediated blood-brain barrier disruption for targeted drug delivery in the central nervous system. Adv Drug Deliv Rev 72:94–109. 24462453 10.1016/j.addr.2014.01.008 PMC4041837

[CIT0002] Aryal M, Vykhodtseva N, Zhang Y-Z, et al (2015). Multiple sessions of liposomal doxorubicin delivery via focused ultrasound mediated blood-brain barrier disruption: a safety study. J Control Release 204:60–9. 25724272 10.1016/j.jconrel.2015.02.033 PMC4385501

[CIT0003] Basu A, Li X, Leong SS. (2011). Refolding of proteins from inclusion bodies: rational design and recipes. Appl Microbiol Biotechnol 92:241–51. 21822901 10.1007/s00253-011-3513-y

[CIT0004] Beenken A, Mohammadi M. (2009). The FGF family: biology, pathophysiology and therapy. Nat Rev Drug Discov 8:235–53. 19247306 10.1038/nrd2792 PMC3684054

[CIT0005] Correia AS. (2007). Fibroblast growth factor-20 increases the yield of midbrain dopaminergic neurons derived from human embryonic stem cells. Front Neuroanat 1:4. 18958198 10.3389/neuro.05.004.2007 PMC2525922

[CIT0006] Grothe C, Timmer M, Scholz T, et al (2004). Fibroblast growth factor-20 promotes the differentiation of Nurr1-overexpressing neural stem cells into tyrosine hydroxylase-positive neurons. Neurobiol Dis 17:163–70. 15474354 10.1016/j.nbd.2004.07.007

[CIT0007] Hartmann A, Hunot S, Michel PP, et al (2000). Caspase-3: a vulnerability factor and final effector in apoptotic death of dopaminergic neurons in Parkinson’s disease. Proc Natl Acad Sci USA 97:2875–80. 10688892 10.1073/pnas.040556597 PMC16023

[CIT0008] Itoh N, Ohta H. (2013). Roles of FGF20 in dopaminergic neurons and Parkinson’s disease. Front Mol Neurosci 6:15. 23754977 10.3389/fnmol.2013.00015 PMC3668169

[CIT0009] Jia X, Tian H, Tang L, et al (2015). High-efficiency expression of TAT-bFGF fusion protein in *Escherichia coli* and the effect on hypertrophic scar tissue. PLoS One 10:e0117448. 25706539 10.1371/journal.pone.0117448 PMC4338132

[CIT0010] Kobus T, Zervantonakis IK, Zhang Y, et al (2016). Growth inhibition in a brain metastasis model by antibody delivery using focused ultrasound-mediated blood-brain barrier disruption. J Control Release 238:281–8. 27496633 10.1016/j.jconrel.2016.08.001 PMC5014601

[CIT0011] Konofagou EE. (2012). Optimization of the ultrasound-induced blood-brain barrier opening. Theranostics 2:1223–37. 23382778 10.7150/thno.5576 PMC3563154

[CIT0012] Liu X, Chen Y, Wu X, et al (2012). SUMO fusion system facilitates soluble expression and high production of bioactive human fibroblast growth factor 23 (FGF23). Appl Microbiol Biotechnol 96:103–11. 22249722 10.1007/s00253-011-3864-4 PMC7080044

[CIT0013] Middelberg AP. (2002). Preparative protein refolding. Trends Biotechnol 20:437–43. 12220907 10.1016/s0167-7799(02)02047-4

[CIT0014] O’Reilly MA, Waspe AC, Ganguly M, et al (2011). Focused-ultrasound disruption of the blood-brain barrier using closely-timed short pulses: influence of sonication parameters and injection rate. Ultrasound Med Biol 37:587–94. 21376455 10.1016/j.ultrasmedbio.2011.01.008 PMC3062725

[CIT0015] Ohmachi S, Mikami T, Konishi M, et al (2003). Preferential neurotrophic activity of fibroblast growth factor-20 for dopaminergic neurons through fibroblast growth factor receptor-1c. J Neurosci Res 72:436–43. 12704805 10.1002/jnr.10592

[CIT0016] Ohmachi S, Watanabe Y, Mikami T, et al (2000). FGF-20, a novel neurotrophic factor, preferentially expressed in the substantia nigra pars compacta of rat brain. Biochem Biophys Res Commun 277:355–60. 11032730 10.1006/bbrc.2000.3675

[CIT0017] Pan J, Li H, Wang Y, et al (2012). Fibroblast growth factor 20 (FGF20) polymorphism is a risk factor for Parkinson’s disease in Chinese population. Parkinsonism Relat Disord 18:629–31. 22342445 10.1016/j.parkreldis.2012.01.017

[CIT0018] Park E-J, Zhang Y-Z, Vykhodtseva N, et al (2012). Ultrasound-mediated blood-brain/blood-tumor barrier disruption improves outcomes with trastuzumab in a breast cancer brain metastasis model. J Control Release 163:277–84. 23000189 10.1016/j.jconrel.2012.09.007 PMC3502612

[CIT0019] Patel MM, Patel BM. (2017). Crossing the blood-brain barrier: recent advances in drug delivery to the brain. CNS Drugs 31:109–33. 28101766 10.1007/s40263-016-0405-9

[CIT0020] Schober A. (2004). Classic toxin-induced animal models of Parkinson's disease: 6-OHDA and MPTP. Cell Tissue Res 318:215–24. 15503155 10.1007/s00441-004-0938-y

[CIT0021] Shimada H, Yoshimura N, Tsuji A, et al (2009). Differentiation of dopaminergic neurons from human embryonic stem cells: modulation of differentiation by FGF-20. J Biosci Bioeng 107:447–54. 19332307 10.1016/j.jbiosc.2008.12.013

[CIT0022] Sleeman IJ, Boshoff EL, Duty S. (2012). Fibroblast growth factor-20 protects against dopamine neuron loss in vitro and provides functional protection in the 6-hydroxydopamine-lesioned rat model of Parkinson’s disease. Neuropharmacology 63:1268–77. 22971544 10.1016/j.neuropharm.2012.07.029

[CIT0023] Sun XW, Wang XH, Yao YB. (2014). Co-expression of Dsb proteins enables soluble expression of a single-chain variable fragment (scFv) against human type 1 insulin-like growth factor receptor (IGF-1R) in *E. coli*. World J Microbiol Biotechnol 30:3221–7. 25256416 10.1007/s11274-014-1749-2

[CIT0024] Tatton WG, Chalmers-Redman R, Brown D, et al (2003). Apoptosis in Parkinson's disease: signals for neuronal degradation. Ann Neurol 53(Suppl. 3):S61–S70,discussion S70–2. 12666099 10.1002/ana.10489

[CIT0025] Thisse B, Thisse C. (2005). Functions and regulations of fibroblast growth factor signaling during embryonic development. Dev Biol 287:390–402. 16216232 10.1016/j.ydbio.2005.09.011

[CIT0026] Thuret S, Bhatt L, O'Leary DDM, et al (2004). Identification and developmental analysis of genes expressed by dopaminergic neurons of the substantia nigra pars compacta. Mol Cell Neurosci 25:394–405. 15033168 10.1016/j.mcn.2003.11.004

[CIT0027] Tian X-Q, Ni X-W, Xu H-L, et al (2017). Prevention of doxorubicin-induced cardiomyopathy using targeted MaFGF mediated by nanoparticles combined with ultrasound-targeted MB destruction. Int J Nanomed 12:7103–19. 10.2147/IJN.S145799 PMC5627735 29026304

[CIT0028] Tian H, Zhao Y, Chen N, et al (2016). High production in *E. coli* of biologically active recombinant human fibroblast growth factor 20 and its neuroprotective effects. Appl Microbiol Biotechnol 100:3023–34. 26603761 10.1007/s00253-015-7168-y

[CIT0029] Tolleson CM, Fang JY. (2013). Advances in the mechanisms of Parkinson's disease. Discov Med 15:61–6. 23375015

[CIT0030] Vlachos F, Tung YS, Konofagou EE. (2010). Permeability assessment of the focused ultrasound-induced blood-brain barrier opening using dynamic contrast-enhanced MRI. Phys Med Biol 55:5451–66. 20736501 10.1088/0031-9155/55/18/012 PMC4005850

[CIT0031] Walkinshaw G, Waters CM. (1994). Neurotoxin-induced cell death in neuronal PC12 cells is mediated by induction of apoptosis. Neuroscience 63:975–87. 7535401 10.1016/0306-4522(94)90566-5

[CIT0032] Wang H, Xiao Y, Fu L, et al (2010). High-level expression and purification of soluble recombinant FGF21 protein by SUMO fusion in *Escherichia coli*. BMC Biotechnol 10:14. 20163718 10.1186/1472-6750-10-14 PMC2831817

[CIT0033] Zhu G, Chen G, Shi L, et al (2015). PEGylated rhFGF-2 conveys long-term neuroprotection and improves neuronal function in a rat model of Parkinson’s disease. Mol Neurobiol 51:32–42. 24930088 10.1007/s12035-014-8750-5

