# Supplementary material for: Efficient treatment of Parkinson’s disease using ultrasonography-guided rhFGF20 proteoliposomes
Source: Drug Deliv. 2018 Jul 25;25(1):1560–9. doi: 10.1080/10717544.2018.1482972 (PMC6060384; doi:10.1080/10717544.2018.1482972)
Supplement: Supplementary Figure S1 [file IDRD_A_1482972_SM5570.docx]

**Supplementary Information**


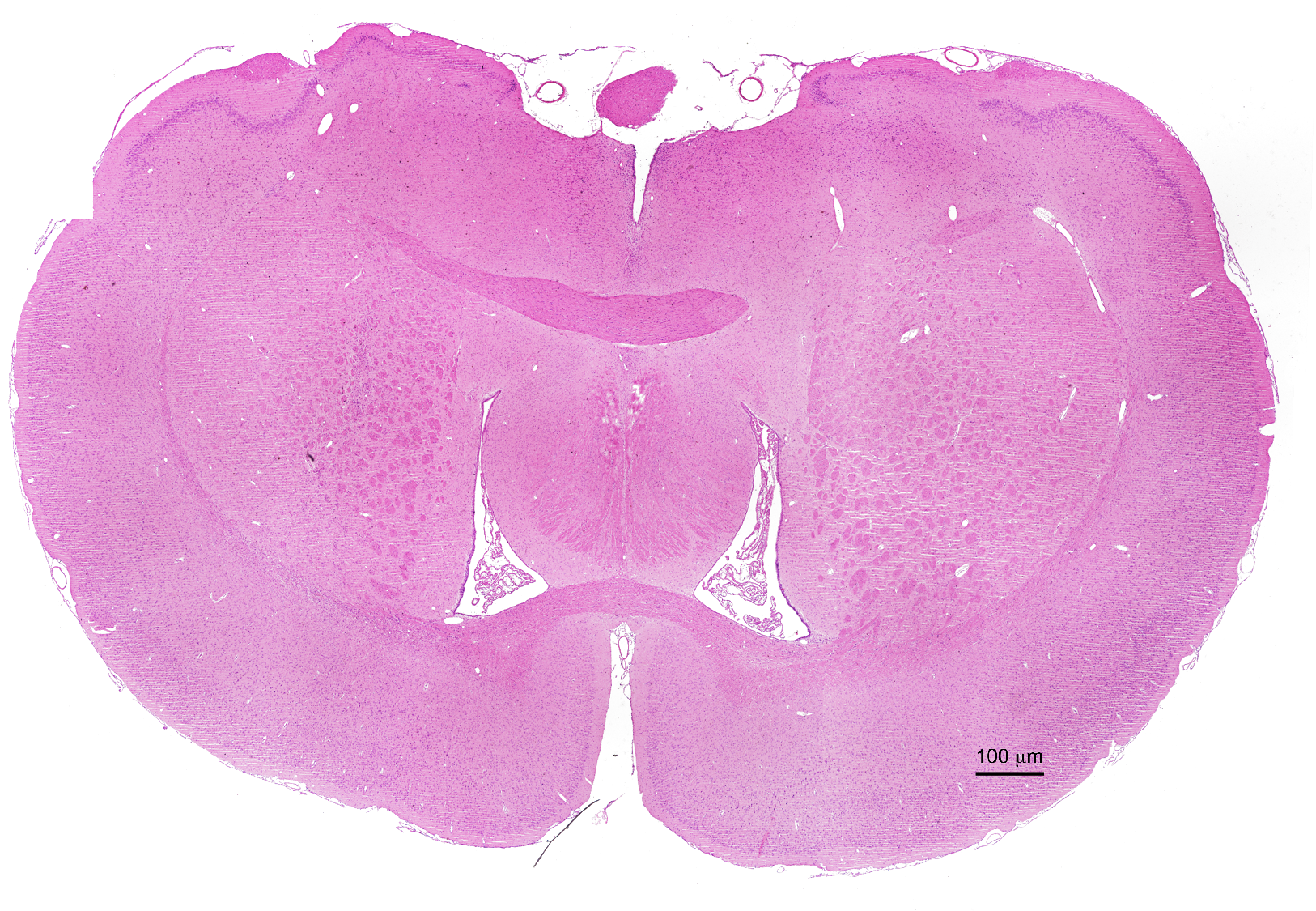


**Figure S1.** Histological examination of the whole brain using haematoxylin and eosin stain (H&E stain). The ultrasound treatment without any therapeutics didn’t cause any extravasation of red blood cells.
